# Three-Dimensional Distribution of Sensory Stimulation-Evoked Neuronal Activity of Spinal Dorsal Horn Neurons Analyzed by *In Vivo* Calcium Imaging

**DOI:** 10.1371/journal.pone.0103321

**Published:** 2014-08-06

**Authors:** Kazuhiko Nishida, Shinji Matsumura, Wataru Taniguchi, Daisuke Uta, Hidemasa Furue, Seiji Ito

**Affiliations:** 1 Department of Medical Chemistry, Kansai Medical University, Hirakata, Osaka, Japan; 2 Pain Research Center, Kansai University of Health Sciences, Kumatori, Osaka, Japan; 3 Department of Information Physiology, National Institute for Physiological Sciences, Okazaki, Aichi, Japan; University of Wurzburg, Germany

## Abstract

The spinal dorsal horn comprises heterogeneous populations of interneurons and projection neurons, which form neuronal circuits crucial for processing of primary sensory information. Although electrophysiological analyses have uncovered sensory stimulation-evoked neuronal activity of various spinal dorsal horn neurons, monitoring these activities from large ensembles of neurons is needed to obtain a comprehensive view of the spinal dorsal horn circuitry. In the present study, we established *in vivo* calcium imaging of multiple spinal dorsal horn neurons by using a two-photon microscope and extracted three-dimensional neuronal activity maps of these neurons in response to cutaneous sensory stimulation. For calcium imaging, a fluorescence resonance energy transfer (FRET)-based calcium indicator protein, Yellow Cameleon, which is insensitive to motion artifacts of living animals was introduced into spinal dorsal horn neurons by *in utero* electroporation. *In vivo* calcium imaging following pinch, brush, and heat stimulation suggests that laminar distribution of sensory stimulation-evoked neuronal activity in the spinal dorsal horn largely corresponds to that of primary afferent inputs. In addition, cutaneous pinch stimulation elicited activities of neurons in the spinal cord at least until 2 spinal segments away from the central projection field of primary sensory neurons responsible for the stimulated skin point. These results provide a clue to understand neuronal processing of sensory information in the spinal dorsal horn.

## Introduction

Spinal dorsal horn (SDH) neurons not only relay sensory information to higher brain centers, but also form neuronal circuits to process primary sensory information [1]. Sensory stimulation-evoked neuronal activity of SDH projection neurons is modified by polysynaptic sensory inputs through interneurons. The fact that pharmacological inhibition and targeted disruption of SDH interneurons disturb somatic sensation indicates crucial roles of the SDH interneurons for neuronal processing of sensory information [2]–[7]. The significance of SDH interneurons is also exemplified by allodynia or hyperalgesia, which are caused at least in part by dysfunction of or damage to these interneurons [8]. SDH interneurons are also involved in the spatial tuning of the tactile and nociceptive systems [9], [10].

Previous electrophysiological and immunohistochemical studies have elucidated neuronal connectivity of SDH projection neurons and interneurons [1]. However, SDH interneurons with various morphology and neurochemical markers interconnect each other to form highly complex circuits, hampering our understanding as to how these heterogeneous neurons cooperate together to process sensory information. Monitoring the pattern of neuronal activity of a large ensemble of SDH neurons would provide data for a comprehensive view about SDH circuits. Since central terminals of primary sensory neurons are spatially organized according to their response profile and their peripheral projection field, analysis of the global distribution pattern of SDH neuronal activity in response to sensory stimulation would uncover how sensory information of primary afferents propagates to the surrounding area by SDH circuits. *In vivo* calcium imaging is a promising technique to monitor activities of many neurons in a single animal, as it overcomes technical limitations of electrophysiological studies [11]. Several investigators have reported *in vivo* calcium imaging of SDH neurons [12]–[14]. Helmchen’s group and Cote’s group recently devised the way to minimize motion artifacts of the SDH during calcium imaging by mechanical stabilization, ratiometric imaging, and the movement compensation device, allowing stable measurement of neuronal activity [15], [16]. However these studies did not focus on the distribution of the recorded neurons.

In the present study, we performed *in vivo* calcium imaging of SDH neurons by using a two-photon microscope to analyze the global distribution pattern of SDH neuronal activity in response to sensory stimulation. For introduction of calcium indicator proteins, we took advantage of *in utero* electroporation, which enables stable expression of calcium indicators in the SDH along a wide area across the rostrocaudal axis [17]. Moreover, the usage of a fluorescence resonance energy transfer (FRET)-based ratiometric calcium indicator protein drastically decreases motion artifacts during calcium recordings [18]. Based on these technological backgrounds, we succeeded in monitoring the activities of multiple SDH neurons at a single cell resolution across a wide region localized 1.4 mm along the rostrocaudal axis and 150 µm in depth. Moreover, we determined the three-dimensional localization of the recorded neurons and analyzed its relationship with their response profile.

## Materials and Methods

### Ethics statement

The animal protocol was approved by the Animal Experimentation Committee of Kansai Medical University (Permit Number: 13-040(01)). All surgery was performed under pentobarbital (50 mg/kg) or urethane (1.2–1.5 g/kg) anesthesia and all efforts were made to minimize suffering.

### Vectors

Construction of *pCAG-EGFP* and *pCAG-mCherry* was described previously [19], [20]. The coding region of *YC-Nano50* was subcloned into the *pCAGMCS* vector to obtain *pCAG-YC-Nano50* [18], [21], [22].

### *In utero* electroporation

*In utero* electroporation was performed as described previously [17]. Briefly, pregnant ICR mice carrying E12.5 embryos (Shimizu Laboratory Supplies Co., Kyoto, Japan) were deeply anesthetized with pentobarbital (50 mg/kg) prior to electroporation. Plasmid DNA was introduced into the central canal of the spinal cord of the embryos by a microinjector (IM-31; Narishige, Tokyo, Japan). Half-ring-type electrodes were attached to the uterus, and 5 electric pulses (35 V, 50 ms) were applied with an electroporator (CUY21SC; Nepagene, Ichikawa, Japan). All animal experiments were approved by the Animal Experimentation Committee of Kansai Medical University.

### *In vivo* calcium imaging

YC-expressing mice of either sex (8–10 weeks of age) were anesthetized with urethane (1.2–1.5 g/kg). The lumbar spinal cord at the level of L1 was exposed by laminectomy. Each mouse was fixed in a stereotactic frame by attaching custom-made clamps to the vertebral column (Narishige). After removal of the dura, agar and cover glass slides were embedded onto the spinal dorsal horn to restrict its movement.

Prior to mechanical and thermal stimulation, hair of the left abdomen was removed by using an electric shaver, and stimulation sites on the skin were marked. Mechanical stimulation was applied by pinching with a forceps for 3 s or brushing for 1 s. For thermal stimulation, we used a custom-made thermal stimulator fashioned from a radiant heat lamp (Hirakata Techno College, Hirakata, Japan). Heat stimulation was applied for 30 s from a baseline temperature of about 30°C. After application of heat stimulation, the skin temperature reached 45°C in 5 s and did not increase above 50°C during the stimulation.

For calcium imaging, images were captured every 0.43 s per recording frame (256 pix×256 pix, 509 µm×509 µm) with an upright two-photon laser-scanning microscope (FV1000 MPE; Olympus, Tokyo, Japan) equipped with a 25×water-immersion objective (XLPlan N; NA of 1.05, Olympus). In a single recording frame, calcium imaging was performed at different confocal planes (every 10–20 µm). The Ti:sapphire laser (MaiTai Deep See; Spectra Physics, Tokyo, Japan) used for excitation was tuned to 850 nm. Emitted fluorescence was short-pass filtered (460–500 and 520–560 nm for CFP and YFP moieties of YC-Nano50 fluorescence, respectively), and detected by photomultipliers. For data analysis, the YFP to CFP fluorescence ratio (R) was filtered with a 1.3-s window. The baseline YFP/CFP ratio (Ro) was calculated as the mean ratio of a 5-s time window immediately before stimulus onset. Peak amplitude was defined as the maximum value of a 10-s window after the stimulus onset for pinch and brush stimuli, or the maximum value during stimulation for heat stimuli. The change in ratio (ΔR/Ro) in response to sensory stimulation was calculated from the peak and the baseline ratio.

### Analysis of three-dimensional distribution of recorded neurons

Before calcium imaging, high-resolution fluorescent images of the calcium recording frame were captured by a two-photon microscope every 2 µm in depth (1024 pix×1024 pix, 509 µm×509 µm). The precise location of cell bodies of recorded neurons was determined from high-resolution images processed by ImageJ. Z coordinates of YC-positive cell bodies were defined as those of a central plane between the dorsal and ventral edge of YC-positive neurons. X and Y coordinates of YC-positive cell bodies were defined as a centroid of YC-positive areas in the pre-determined Z plane. In some cases, calcium responses of YC-positive neurons were obtained in 2 neighboring planes because of a strong YC signal. We carefully compared 2 neighboring images to prevent counting such neurons as 2 independent neurons.

To describe the three-dimensional distribution of recorded neurons, we reconstructed the location of their cell bodies in serial transverse sections. The dorsal outline of the grey matter in each transverse section was also determined from high-resolution images. Since YC-positive superficial SDH neurons extended many processes, we could easily recognize the boundary between YC-fluorescent-positive and -negative areas in high-resolution images, and defined the boundary as the dorsal edge of the grey matter. The location of these boundaries in high-resolution images was traced and described in reconstructed three-dimensional images. For categorization of recorded neurons regarding their depth, we drew lines parallel to the dorsal outline at 50 µm intervals, and counted the number of neurons in each range of depth in the SDH. The percentage of responsive neurons was calculated from the number of responsive neurons and recorded neurons within each range of depth (0–50 µm, 50–100 µm, 100–150 µm). Cells with calcium transients whose ΔR/Ro was equal to or above 15% were defined as responsive neurons.

### Retrograde labeling of sensory neurons by use of Cholera toxin B

Eight-week-old ICR mice of either sex were deeply anesthetized with pentobarbital (50 mg/kg). Prior to injection of Cholera toxin B (CTB), hair was removed using an electric shaver. P1 was defined as a point around the posterior abdomen whose pinch stimulation efficiently evoked calcium transients of SDH neurons at the L1 level. For CTB injection, P1 was re-defined by anthropometric landmarks such as underlying bone prominences located in the lateral border of the trunk along the dorsoventral axis and at 1 cm rostral to the root of the hindlimb along the rostrocaudal axis. Two microliters of Alexa488- or Alexa555-conjugated CTB (1 mg/ml; Invitrogen, Carlsbad, CA, USA) was loaded into a fine glass capillary and injected subcutaneously into the left abdomen by a microinjector (IM-31, Narishige). The mice were perfused 7 days after the injection. Fluorescent images of the labeled cell bodies in the DRG were taken by a camera attached to a fluorescence microscope (Axioplan2; Carl Zeiss, Jena, Germany). For the detection of primary sensory afferents in the SDH, 50-µm serial transverse sections were prepared by use of a cryostat and fluorescent images were obtained by a confocal microscope (LSM510, Carl Zeiss). The rostrocaudal distance of CTB-positive central terminals or that between central terminals was determined by counting the number of serial transverse sections of the spinal cord.

### Histochemistry

Sixteen-micrometer transverse sections of the thoracic spinal cord electroporated with *pCAG-EGFP* or *pCAG-mCherry* were Nissl-stained (NeuroTrace 530/615, Invitrogen) or immunostained with rabbit anti-Lmx1b polyclonal antibody [23], rabbit anti-Pax2 polyclonal antibody (Invitrogen), mouse anti-Calbindin monoclonal antibody (Swant, Marly, Switzerland), mouse anti-nNOS monoclonal antibody (BD Biosciences, San Jose, CA, USA), mouse anti-NeuN monoclonal antibody (Millipore, Billerica, MA, USA), or mouse anti-GFAP monoclonal antibody (Millipore). These sections were further incubated with Alexa 488- or Alexa 546-conjugated anti-rabbit or anti-mouse antibody (Invitrogen), and their fluorescence was observed with a confocal microscope (LSM510; Carl Zeiss). For determination of laminae I, II, and III, transverse sections of thoracic spinal cords of P21 mice or L1 spinal cord of 8-week-old mice were Nissl-stained. Lamina boundaries were determined based on previously established criteria [24]. Thickness of each lamina was measured in the lateral part of the spinal cord where the *in vivo* calcium imaging was usually performed.

## Results

### Gene transfer of a genetically encoded calcium indicator by *in* *utero* electroporation

**Figure 1:**
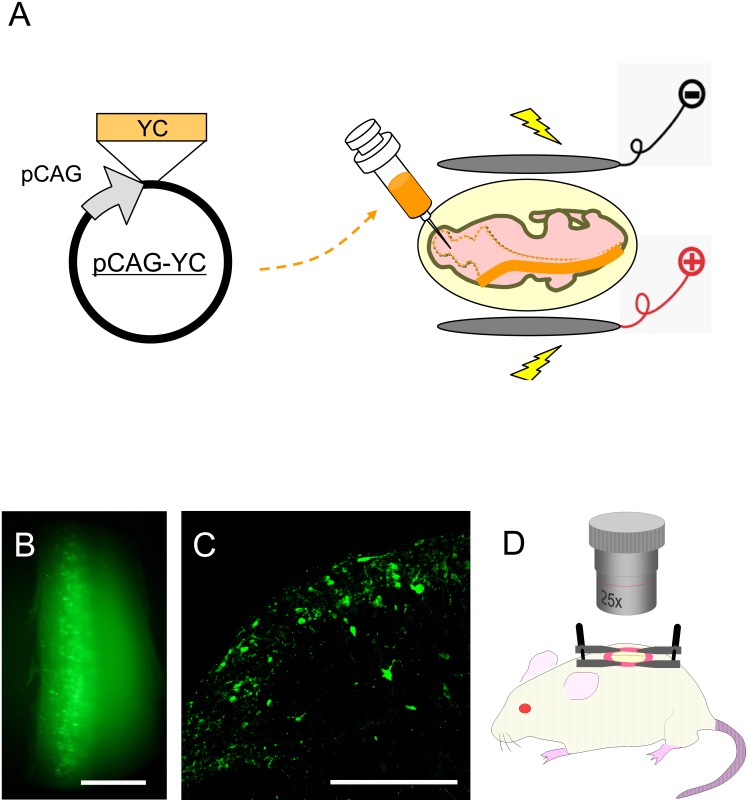
Methodological overview. (A) *In utero* electroporation of Yellow Cameleon expression vector. *pCAG-YCnano50* was injected into the spinal cord central canal at E12.5 and electric pulses were applied with an electroporator. (B) YC expression in the spinal dorsal horn of an 8-week-old mouse. YC was expressed in the left side of the spinal cord around the L1 level. Scale bar, 1 mm. (C) Transverse section of YC-expressing SDH at L1. SDH on the left side is shown. Scale bar, 200 µm. (D) *In vivo* calcium imaging of SDH neurons was performed in YC-expressing mice (8–10 weeks old). The spinal cord at the level of L1 was exposed by laminectomy. The mouse was fixed in a stereotactic frame by attaching custom-made clamps to the vertebral column, and calcium imaging was performed by use of a two-photon microscope.

**Figure 2:**
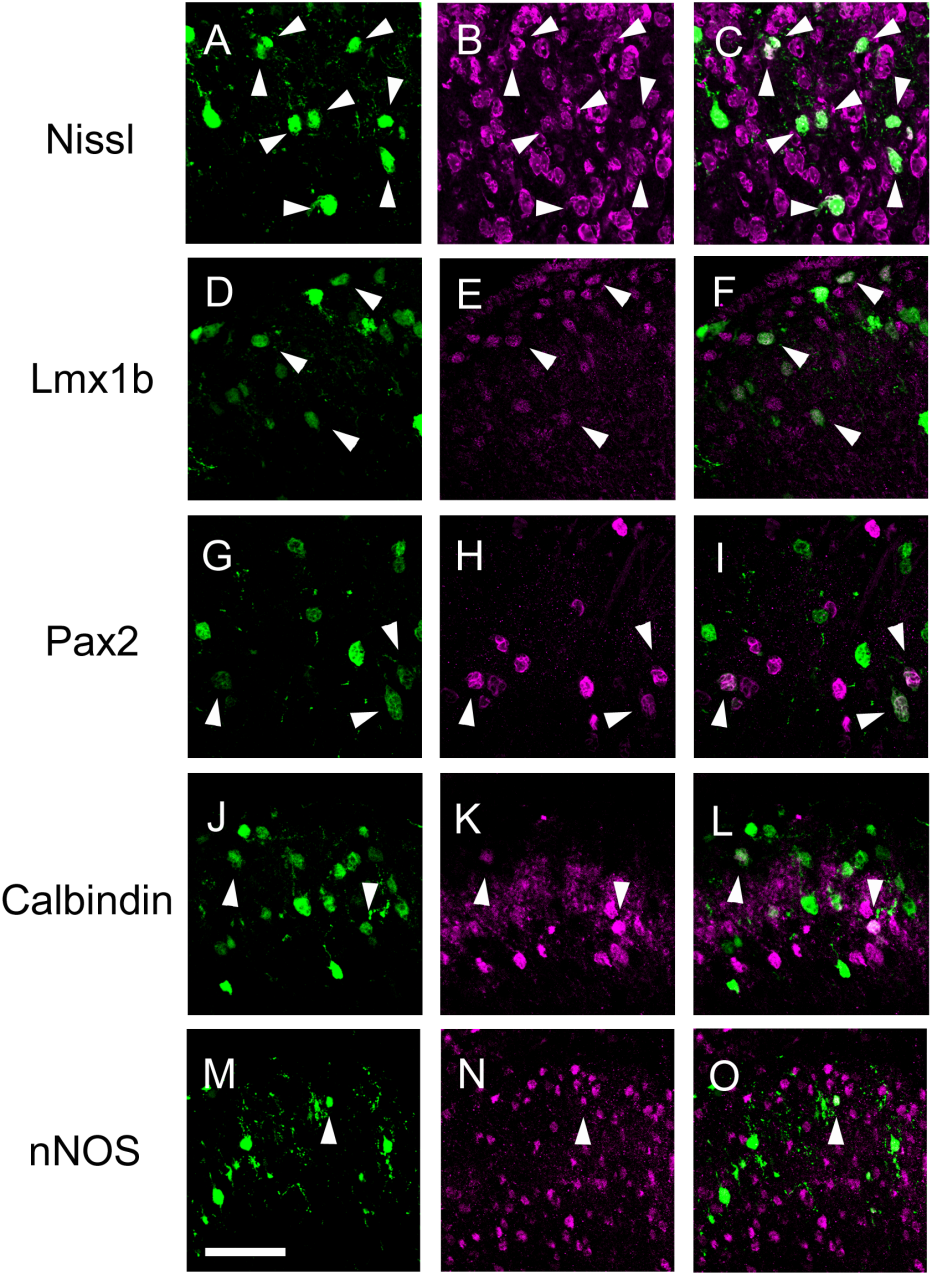
Identity of EGFP-expressing cells in the SDH. *pCAG-EGFP* was electroporated into the spinal cord at E12.5, and transverse sections were prepared from the thoracic spinal cord of the electroporated mice at P21. The sections were Nissl-stained (B, C) or immunostained with anti-Lmx1b (E, F), anti-Pax2 antibody (H, I), anti-Calbindin antibody (K, L), or anti-nNOS antibody (N, O). EGFP fluorescence (A, C, D, F, G, I, J, L, M, O), and immunofluorescence of anti-Lmx1b (E, F), anti-Pax2 (H, I), anti-Calbindin (K, L), and anti-nNOS (N, O) are shown. Arrowheads indicate double-positive cells. Scale bar, 50 µm.

For introduction of calcium indicators, we performed gene transfer by an expression vector encoding a genetically encoded calcium indicator protein, YC-Nano50, by performing *in utero* electroporation (Fig. 1A). Since birthdate analysis using BrdU showed that superficial SDH cells are born around E12.5 (Fig. S1), we chose to perform *in utero* electroporation of E12.5 embryos. YC expression in electroporated adult mice was found across a broad area of the SDH along the rostrocaudal axis (Fig. 1B). YC-positive cells were mainly localized in the superficial SDH, as expected from our birthdate analysis (Fig. 1C). The identity of labeled cells was analyzed after electroporation with an EGFP or mCherry expression vector. Most labeled cells in the superficial SDH (Rexed’s lamina I–III) were Nissl-positive (98.4±0.9%, 5 mice, 978 cells) and NeuN positive (92.9±2.4%, 6 mice, 383 cells), but not GFAP positive (6/190, 2 mice), suggesting that our *in utero* electroporation procedure labeled mostly superficial SDH neurons (Fig. 2A–C, Fig. S2). On the other hand, 4.1±2.1%, 7.6±3.6%, and 4.2±1.7% of Nissl-positive cells were EGFP-positive within laminae I, II, and III, respectively, suggesting that a small subset of cells could be labeled by this method (n = 5 mice, lamina I:2136 cells; lamina II:6796 cells; lamina III:8076 cells). We further analyzed the subtype identity of these EGFP-positive neurons in the superficial SDH by immunostaining with antibodies against Lmx1b and Pax2, which are markers for excitatory and inhibitory interneurons, respectively, in the SDH [23], [25], [26]. Among the EGFP-positive cells in the laminae I–III, 56.6±4.2% (n = 3 mice, 859 cells) were Lmx1b-positive cells, and 19.9±3.1% (n = 3 mice, 628 cells) were Pax2-positive ones (Fig. 2D–I). These results were further confirmed by immunostaining with antibodies against calbindin and nNOS, markers for a subpopulation of excitatory and inhibitory interneurons, respectively [27], [28] (Fig. 2 J–O). It is likely that the majority of labeled neurons were interneurons, since most projection neurons in the superficial SDH constitute only 5% of lamina I neurons [1]. These results suggest that gene transfer by *in utero* electroporation allowed the introduction of the gene into a variety of neuronal subtypes in the superficial SDH.

### *In vivo* calcium imaging

**Figure 3:**
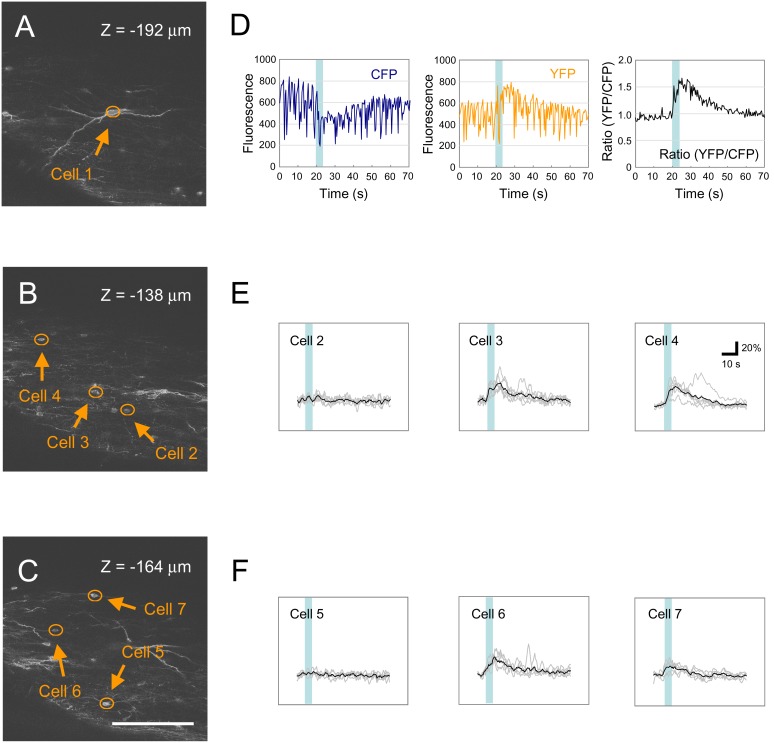
*In vivo* calcium imaging of SDH neurons at L1. (A–C) *in vivo* calcium imaging was performed at 3 different confocal planes. Scale bar, 200 µm. (D) Fluorescent traces of CFP and YFP of Cell 1 are shown in the left and middle panels, respectively. The change in the ratio (YFP/CFP) of Cell 1 is shown in the right panel. Vertical blue bars indicate pinch stimulation applied to the ipsilateral abdomen. (E–F) Changes in the ratios (YFP/CFP) of 6 cells (Cells 2–7) seen in B and C are shown. Ratio traces were smoothed by a 1.3-s moving window. Grey, 6 individual trials; Black, average trace.

**Figure 4:**
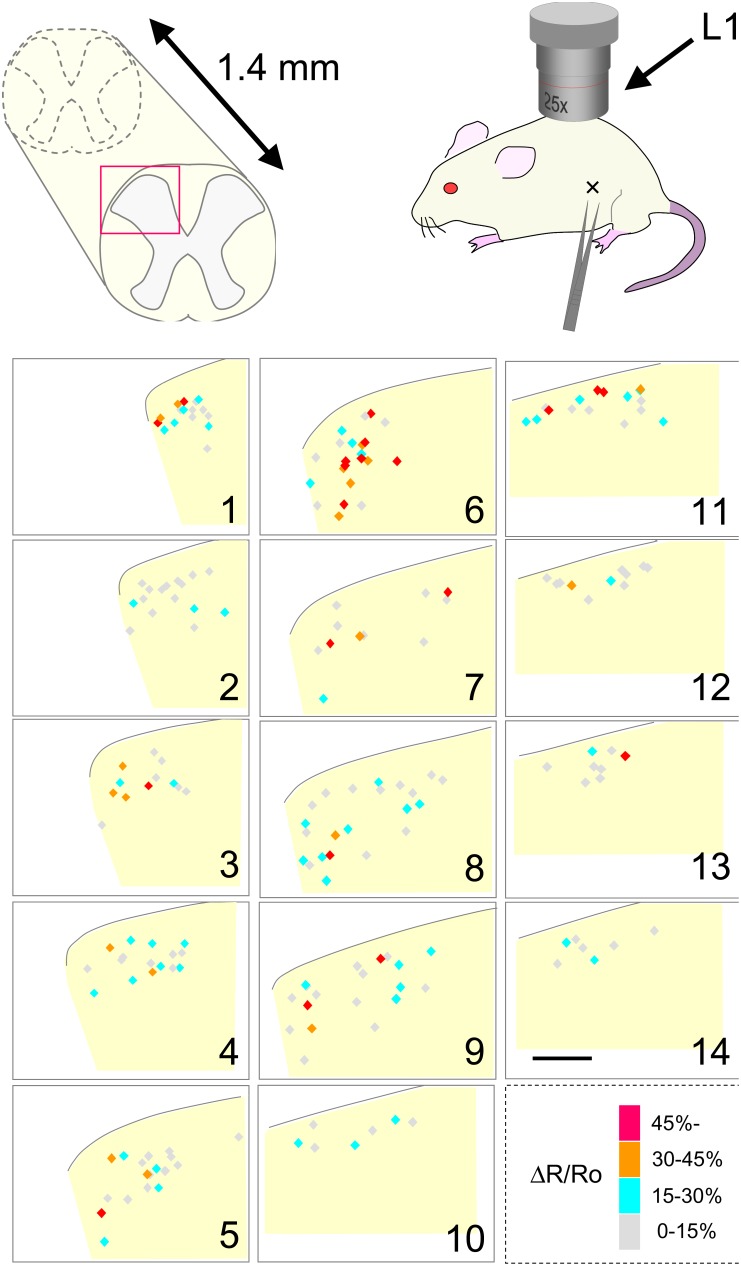
Three-dimensional distribution map of neuronal activity of SDH neurons in response to pinch stimulation. *In vivo* calcium imaging was performed in the SDH at the level of L1 following pinch stimulation of the ipsilateral abdomen. Localization of recorded neurons was reconstructed as serial transverse sections along the rostrocaudal axis. Each section is 100 µm in thickness (sections 1 and 14 are rostral and caudal limits, respectively), and the outline in each section indicates the dorsal boundary of the grey matter of the SDH determined from high-resolution images of the recorded area (see Materials and Methods). Cell bodies of recorded neurons (202 cells) are shown as diamonds, and they are color-coded according to the amplitude of calcium transients in response to pinch stimulation. Red, >45%; Yellow, 30–45%; Blue, 15–30%; Grey, 0–15%. Scale, 100 µm.

We then performed *in vivo* calcium imaging of SDH neurons of 8- to 10-week-old mice electroporated with the YC expression vector. Based on the dermatome map, calcium responses of SDH neurons at the L1 level were examined following pinch stimulation applied to the posterior portion of the ipsilateral abdomen [29]. Using a two-photon microscope, YC-positive cells could be resolved down to a maximum imaging depth of about 200 µm (Fig. 3). Following cutaneous pinch stimulation, we observed a gradual increase in YFP fluorescence and decrease in CFP fluorescence in some neurons albeit with transient large motion artifacts (Fig. 3D, left and middle). In contrast, the YFP to CFP fluorescence ratio exhibited a drastic reduction in noises owing to the fact that YC being a ratiometric calcium indicator efficiently compensates for a fluctuation in fluorescence-detected calcium responses derived from moving tissue (Fig. 3D, right). Calcium imaging of a stack of consecutive focal planes allowed us to analyze neuronal activity of about 100 SDH neurons within a 150-µm depth from the dorsal edge of the grey matter in a single recording frame. The three-dimensional distribution of recorded neurons was then reconstructed from high-resolution images captured in the same frame (see Materials and Methods). By calcium imaging of 3 neighboring frames along the rostrocaudal level and connecting these images together, we could measure calcium transients of 202 SDH neurons at the L1 level distributed across a 1.4-mm length along the rostrocaudal axis (Fig. 4). Classification of recorded neurons by the amplitude of calcium transients showed that each SDH neuron had a differential responsiveness toward cutaneous pinch stimulation.

### Calcium imaging following different sensory stimulation

**Figure 5:**
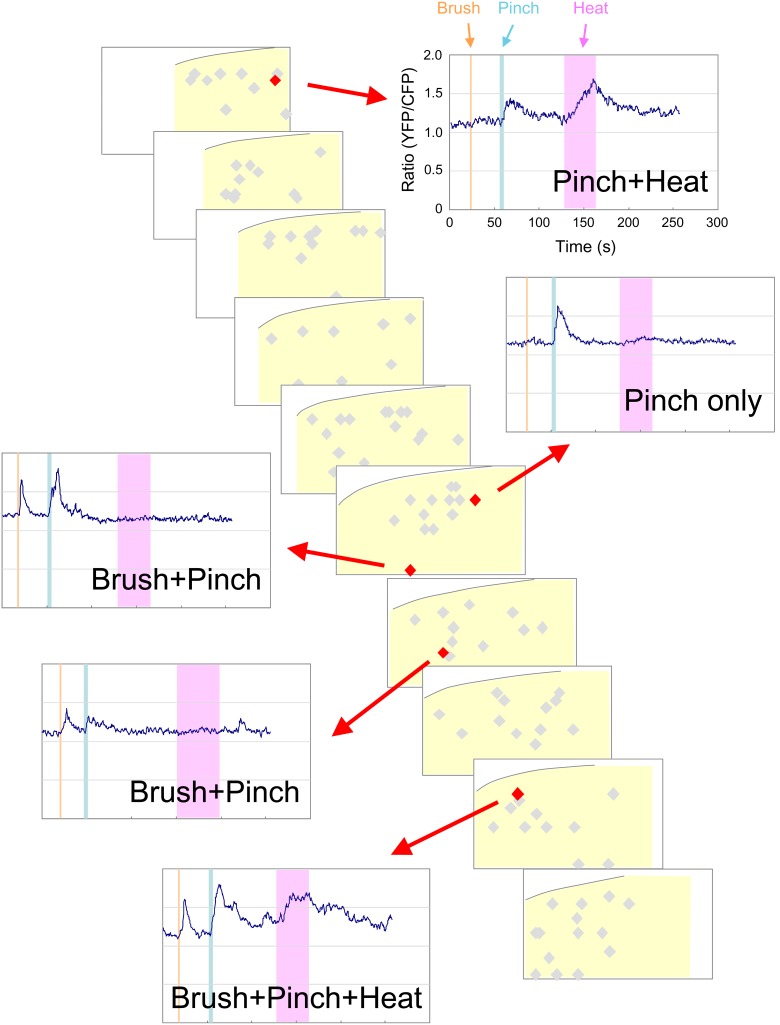
Three-dimensional distribution map of neuronal activity of SDH neurons in response to various stimulants. Brush, pinch, and heat stimulants were sequentially applied to the skin of the ipsilateral abdomen. Localization of recorded neurons (grey diamonds) is shown as serial transverse sections (50 µm in thickness). Each trace represents the change of YFP/CFP ratio of 5 representative cells (red diamonds) in response to individual trial of brush (yellow bar), pinch (blue bar), and heat (pink bar) stimulation.

**Figure 6:**
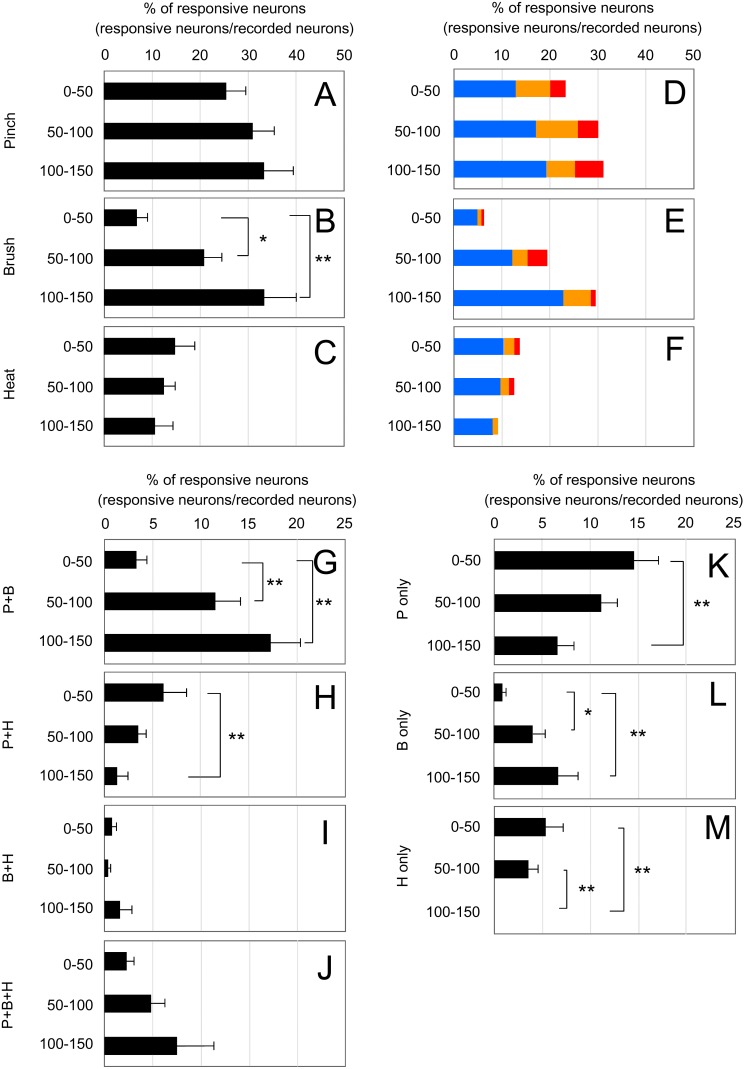
Lamina distribution pattern of SDH neurons in response to pinch, brush, or heat stimulation. Following calcium imaging in response to pinch, brush, or heat stimulation, the depth of each neuron from the dorsal edge of the grey matter was measured. (A–C) Recorded neurons were classified into 3 groups according to their depth: 0–50 µm, 50–100 µm, and 100–150 µm. The percentage of responsive neurons within each depth is shown. (D–F) Total recorded neurons (785 cells) were further classified by the amplitude of their calcium transients. Red: >45%, Yellow: 30–45%, Blue: 15–30%. (G–M) Recorded neurons were classified according to the response profiles of different sensory modalities, and the percentage of responsive neurons within each depth is shown. (G) pinch (+), brush (+), heat (−) neurons. (H) pinch (+), brush (−), heat (+) neurons. (I) pinch (−), brush (+), heat (+) neurons. (J) pinch (+), brush (+), heat (+) neurons. (K) pinch (+), brush (−), heat (−) neurons. (L) pinch (−), brush (+), heat (−) neurons. (M) pinch (−), brush (−), heat (+) neurons. All values are means of 8 mice ± S.E.M. *p<0.05, **p<0.01. Data were analyzed by non-repeated measures ANOVA, and statistical significance was examined by Bonferoni posthoc comparisons (n = 8 mice; 0–50 µm: 349 cells, 50–100 µm: 337 cells, 100–150 µm: 99 cells).

Primary sensory afferents terminate with a lamina-specific distribution in the SDH depending on their subtypes [1]: Central afferents of low-threshold mechanoreceptor C and Aδ fibers terminate at the inner lamina II and lamina III, respectively, whereas those of nociceptive and thermoreceptive C and Aδ fibers terminate at lamina I and outer lamina II [30]–[34]. Analysis of SDH neuronal activities in response to sensory stimulation affords insight into how primary sensory stimulation received by a second-order SDH neuron is transmitted to the neighboring lamina through the trans-laminar connectivity of interneurons. We then examined *in vivo* calcium imaging of SDH neurons in response to innocuous mechanical (Brush) and noxious thermal (Heat) stimulation as well as to noxious mechanical (Pinch) stimulation. Each SDH neuron had differential responsiveness to these different types of sensory stimulation (Fig. 5). Between responsive neurons, there were some differences in the decay kinetics of the calcium transients. In order to analyze overall laminar distribution pattern of responsive neurons, we performed this experiment using several mice to calculate the percentage of responsive neurons within the depth ranges of 0–50 µm, 50–100 µm, and 100–150 µm (Fig. 6A–C, Table S1). The thickness of laminae I, II, and III in the SDH at the level of L1 was 20.3±1.6 µm, 56.5±5.0 µm, and 85.6±1.9 µm, respectively (n = 3 mice, 8 weeks old). Thus, the depth of 0–50 µm, 50–100 µm, and 100–150 µm roughly corresponded to lamina I and outer lamina II, inner lamina II and outer lamina III, and inner lamina III, respectively. Pinch stimulation elicited calcium responses in more SDH neurons than did the 2 other types of stimulation and the laminar distribution of pinch-responsive neurons was almost uniform within the depth of 150 µm (Fig. 6A). Heat-responsive neurons were much less numerous than pinch-responsive neurons, but the laminar distribution pattern of these neurons was similar to that of pinch-responsive neurons (Fig. 6C). In contrast, the brush-responsive neurons in the deeper laminae were significantly more numerous than those in the shallow lamina (0–50 µm: 7.0±2.1%, 50–100 µm: 21.1±4.4%, 100–150 µm: 33.0±6.2%; n = 8 mice; Fig. 6B). Neurons with a certain range of calcium transients did not seem to exhibit a biased laminar distribution (Fig. 6D–F, Table S2). Next, we categorized neurons according to their responsiveness to the 3 types of sensory stimulation and analyzed the laminar distribution of neurons in each category (Fig. 6G–M). More neurons that responded to only pinch (Pinch only), only heat (Heat only) or pinch and heat but not brush (Pinch + Heat) were present in the shallow lamina than in the deeper lamina (Fig. 6H, K, M). In contrast, there were more neurons that responded to only brush (Brush only), or pinch and brush but not heat (Pinch + Brush) localized in the deeper lamina than in the shallow lamina (Fig. 6G, L), similar to the distribution pattern of total brush-responsive neurons. Several percent of neurons constituted neurons responsive to all 3 sensory stimulations (Pinch + Brush + Heat), whereas there were very few neurons responsive to the brush and heat combination (Brush + Heat) in the SDH (Fig. 6J, I). Collectively, monitoring neuronal activity of a large ensemble of SDH neurons revealed a tendency to correlate response profiles of SDH neurons with their laminar location.

### Calcium imaging in response to pinch stimulation toward different points

**Figure 7:**
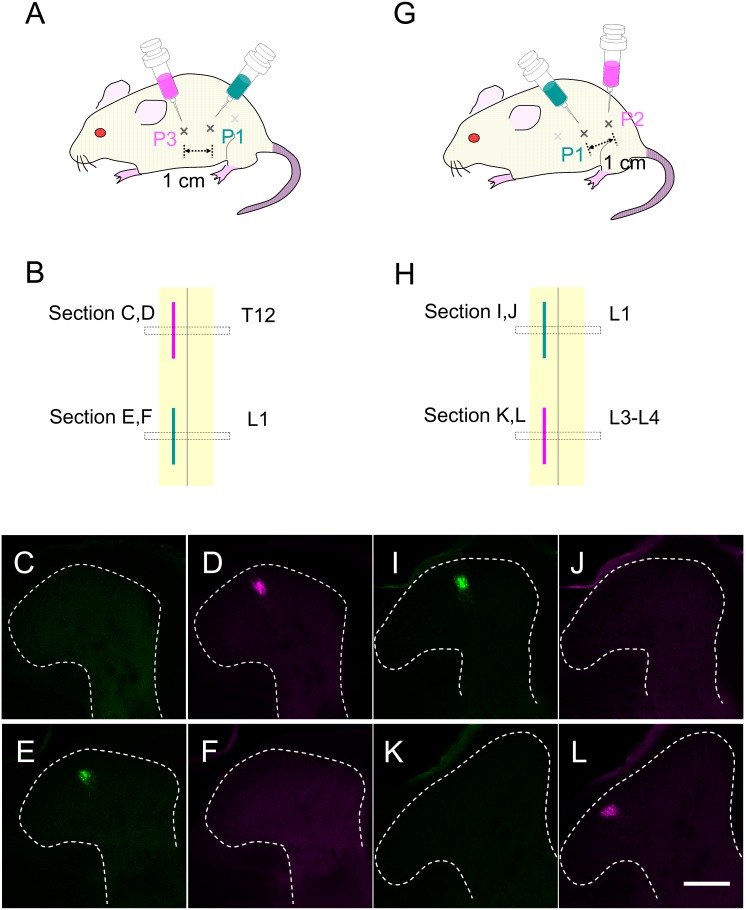
Analysis of the central projection of primary sensory neurons by use of retrograde labeling. (A–F) Alexa488 (green)- and Alexa555 (magenta)-conjugated cholera toxin B (CTB) were respectively injected into P1 and P3 in the abdomen. (A) Seven days later, transverse sections of the spinal cord were prepared. Alexa555-positive and Alexa488-positive central projections were found in the transverse sections around T12 and L1 levels, respectively (C–F). (G–L) Alexa488- and Alexa555-CTB were respectively injected into P1 and P2 in the abdomen. (G) Alexa488- and Alexa555-positive central projections were found in the transverse sections of the spinal cord around L1 and L3 to L4 levels, respectively (I–L). Scale, 100 µm.

SDH interneurons extend dendritic processes and axons preferentially along the rostrocaudal axis [35], raising the possibility that primary sensory inputs in the SDH efficiently propagate along the rostrocaudal axis through SDH interneurons. In order to examine the rostrocaudal spreading of sensory information in the SDH, we next investigated calcium transients of SDH neurons in response to pinch stimulation at 3 different points in the abdomen. We carefully searched for the point around the left abdomen whose pinch stimulation efficiently activated many neurons in the pre-determined recording frame at L1. We designated this point as “P1”. Two additional points of the skin 1 cm caudal and 1 cm rostral to P1 were designated as “P2” and “P3”, respectively. To examine the central projection field of primary sensory neurons responsible for these skin points, we retrogradely labeled primary sensory neurons with Cholera toxin B (CTB), by injecting it into P1, P2, and P3 (Fig. 7). Injection of CTB into P1 preferentially labeled L1-DRG neurons, while its injection into P2 or P3 labeled L3- and L4-DRG or T12-DRG, respectively. The size of CTB-labeled central projection fields in the SDH was less than 50 µm along the mediolateral axis, and about 1.5 mm along the rostrocaudal axis (Fig. 7C–F, I–L). Compared to the previous dermatome analysis using DiI crystal [29], central projection fields analyzed in our study seems to be longer along the rostrocaudal axis, possibly because CTB solution injected into the skin might diffuse around the neighboring area. Central terminals with strong CTB signals were usually found 50–100 µm below the dorsal surface, but weak signals could be seen up to 200 µm in depth, consistent with previous results [30]. Central projection fields of P1 and P2 were 2.0±0.4 mm apart from each other rostrocaudally, and the distance between these centers was 3.3±0.4 mm (n = 3). Similar results were obtained from the mice in which CTB had been injected into P1 and P3 (distance between projection fields: 1.8±0.2 mm, distance between the centers: 3.4±0.3 mm [n = 3]). These results showed that the central projection field of the primary sensory neurons responsible for P1 and the points 1 cm rostrocaudally away from P1 did not overlap and were about 2 mm apart from each other.

**Figure 8:**
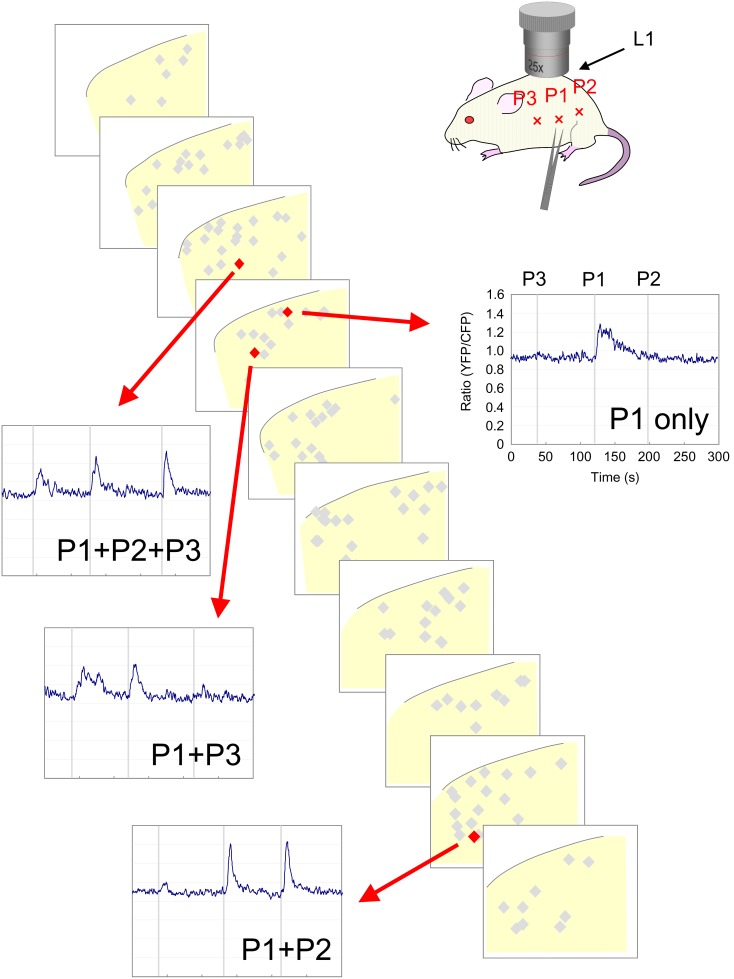
SDH neuronal activity in response to pinch stimulation applied to 3 different points. Pinch stimulation was applied to 3 different points in the ipsilateral abdomen. P2 and P3 were 1-dimensional distribution of recorded neurons (grey diamonds) is shown as serial transverse sections (50 µm in thickness). The YFP/CFP ratio of 4 representative cells (red diamonds) is shown. Each trace represents the change of YFP/CFP ratio of 4 representative cells (red diamonds) in response to individual trial of pinch stimulation to P3 (left bar), P1 (central bar), and P2 (right bar).

**Figure 9:**
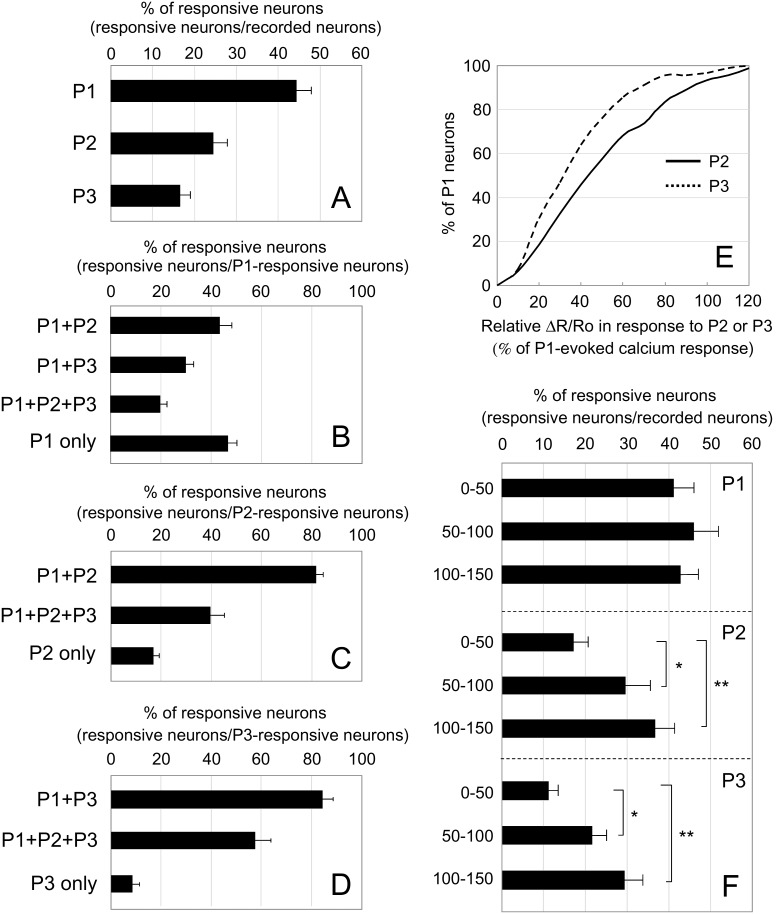
Activation pattern of SDH neurons in response to pinch stimulation applied to 3 different points. Pinch stimulation was applied to 3 different points in the left (ipsilateral) abdomen, as shown in Figure 8. (A) The percentage of responsive neurons among total recorded neurons is shown in the bar graph. All values are means of 14 mice ± S.E.M. (n = 14 mice, 1068 cells). The percentage of neurons that responded to stimulation at P1 (P1), P2 (P2), P3 (P3) is shown. (B–D) The percentage of responsive neurons in each of the specified categories (see graphs) among P1-responsive (B; n = 14 mice, 458 cells), P2-responsive (C; n = 14 mice, 255 cells), and P3-responsive neuronal populations (D; n = 14 mice, 171 cells) is shown. (E) Among P1-responsive neurons (458 cells, 14 mice), 92 cells showed a strong response (ΔR/Ro>45%). The peak amplitudes of P2 and P3 stimulation relative to P1 stimulation among these 92 cells are shown as a cumulative frequency plot. (F) Recorded neurons are classified into 3 groups according to the depth from the dorsal edge of the grey matter: 0–50 µm, 50–100 µm, and 100–150 µm. The percentage of responsive neurons among each group is shown. All values are means of 11 mice ± S.E.M. *p<0.05, **p<0.01. Data were analyzed by non-repeated measures ANOVA, and statistical significance was examined by Bonferoni posthoc comparisons (n = 11 mice; 0–50 µm: 367 cells, 50–100 µm: 352 cells, 100–150 µm: 137 cells).

We then performed *in vivo* calcium imaging of SDH neurons following pinch stimulation at P1, P2, and P3, and then reconstructed the three-dimensional localization of responsive neurons at the L1 level (Fig. 8). Each SDH neuron had differential responsiveness toward pinch stimulation at these 3 points. Analysis of overall response profile by in vivo imaging of several mice revealed that P2 or P3 stimulation elicited calcium transients in quite a few SDH neurons at the L1 level (Fig. 9). P1 stimulation elicited a response in 44.3±3.6% of the SDH neurons, whereas stimulation at P2 and P3 elicited one in 24.4±3.5% and 16.4±2.5%, respectively (Fig. 9A, Table S3; n = 14 mice, 1068 cells). These results suggest that cutaneous pinch stimulation elicited activities of SDH neurons localized rostrocaudally far beyond the central projection field of primary sensory neurons responsible for the stimulated skin point. Among the P1-responsive neuronal population, 46.4±4.0% were activated only by P1, whereas 43.4±4.9%, 29.8±3.1%, and 19.6±3.0% of neurons were also activated by P2, P3, and both P2 and P3, respectively (Fig. 9B, Table S3; n = 14 mice, 458 cells). Interestingly, more than 80% of P2- or P3-responsive neurons were P1-responsive neurons (Fig. 9C, D), suggesting that P2- and P3-responsive neuronal pathway preferentially interacts with P1-responsive neurons at the L1 level. Next, we analyzed the amplitude of P2- or P3-evoked calcium responses in P1-responsive neurons (Fig. 9E, Table S4). In most P1-responsive neurons, calcium transients following P2 or P3 stimulation were weaker than those when P1 was stimulated. In half of the P1-responsive neuronal population, P2- and P3-evoked calcium transients were more than 30% and 40%, respectively, compared with those by P1 stimulation; and in 20% of the P1-responsive neuronal population, they were more than 50% and 70%, respectively, compared with those by P1 stimulation. These results indicate that each P1-responsive neuron had a differential response toward P2 and P3 stimulation. We next analyzed the laminar distribution of P1-, P2-, and P3-responsive neurons (Fig. 9F, Table S5). Interestingly, P2 and P3 stimulation activated more neurons in the deeper laminae than in the shallow lamina, whereas P1-responsive neurons were evenly present across all the laminae. These results indicate that SDH neurons in the deeper laminae receive sensory inputs from wider skin area than those in the shallow laminae.

## Discussion

Since previous electrophysiological studies have examined sensory stimulation-evoked neuronal activity of very few SDH neurons in a single animal, neuronal activity pattern of ensemble of SDH neurons remains unclear. By imaging a large population of SDH neurons *in vivo*, we for the first time clarified the three-dimensional neuronal activity map of SDH neurons in response to cutaneous sensory stimulation. Our results provided a clue to understand how sensory information is processed by SDH neuronal circuits.

### Technical consideration of *in* *vivo* calcium imaging

Gene transfer of calcium indicator proteins by *in utero* electroporation has several advantages for application of *in vivo* calcium imaging. First, calcium indicator proteins can be introduced into SDH neurons across a wide area along the rostrocaudal axis. Recorded neurons included a heterogeneous population of SDH neurons, such as excitatory and inhibitory interneurons (Fig. 2). Second, gene transfer by *in utero* electroporation allows us to keep the spinal cord more intact during calcium recording, preventing from potential tissue damage and inflammatory responses. In comparison, the *in vivo* patch clamp technique requires removal of the arachnoid and/or pia membrane, and *in vivo* calcium imaging using a calcium indicator dye requires its injection into the grey matter [14], [36], [37]. Third, we can immediately evaluate the feasibility of newly updated calcium indicator proteins by the current technique, since improved versions of calcium indicator proteins have been continuously reported year after year. In the present study, the application of the ratiometric calcium indicator protein YC-Nano50 together with mechanical stabilization of the SDH originally devised by Helmchen’s group drastically minimized motion artifacts during *in vivo* calcium imaging [14] (Fig. 3F), which helped precise measurement of calcium responses. In future, employment of more sensitive calcium indicator proteins may help to overcome the limitation of the imaging depth of the two-photon microscope, and enable us to measure more subtle calcium transients in the spine [38].

### Laminar localization pattern of SDH neurons responsive to different sensory stimulation

Woolf and Fitzgerald reported a correlation between the location of neuronal cell bodies and their response profiles based on electrophysiological recording of a small number of SDH neurons in laminae I and II [39]. Here, we addressed this issue by measuring activities of hundreds of SDH neurons localized in laminae I–III by *in vivo* calcium imaging. Brush-responsive neurons were localized in the deeper laminae more than in the shallow lamina. This laminar distribution pattern is similar to that of central terminals of low-threshold mechanoreceptor C and Aδ fibers [30]. In contrast, pinch-responsive neurons were evenly distributed throughout the laminae. However, these neurons do not necessarily comprise only “nociceptive neurons”, since pinching also induces touch and stretch stimulation of the skin [40]. Thus, it is highly possible that some subpopulation of the pinch-responsive neurons was sensitive only to innocuous mechanical stimulation. “Pinch + Brush” neurons would be the candidate of such neurons since their laminar localization pattern was similar to that of “Brush only” neurons. If the assumption above would be correct, then “nociceptive neurons” may be localized in the shallow lamina more than in the deeper laminae, similar to the pattern of “Pinch only” neurons.

The laminar localization pattern of brush-responsive neurons and presumably “nociceptive neurons” suggests that sensory stimulations preferentially evoke neuronal activities of SDH neurons in the lamina where central afferents of the primary sensory neurons terminate. This may be partially because most second-order SDH neurons receive primary sensory afferents in the lamina where their cell bodies reside. In lamina II, dendrites of most SDH interneurons are elongated in the rostrocaudal direction with limited dorsoventral spread [35]. Also, sensory information received by second-order SDH neurons does not seem to be freely propagated to the neighboring lamina through the trans-laminar neuronal network, as supported by the morphology and the connectivity of SDH interneurons. Axonal arbors of lamina II excitatory interneurons are always found in lamina II, but not always in laminae I and III [41]. In addition, excitatory inputs of SDH neurons arise from a wider rostrocaudal area than a dorsoventral area [42].

### Responsiveness of SDH neurons to multiple sensory stimulations

We found quite a few SDH neurons that responded to more than 1 type of sensory stimulation, such as “Pinch + Brush”, “Pinch + Heat”, and “Pinch + Brush + Heat” neurons, which were qualitatively similar to those reported in previous electrophysiological studies in the rat [36], [37], [39], [43]. Sensitivity of SDH neurons to multiple sensory stimulations may be partially due to responsiveness of primary sensory neurons. As discussed above, some subpopulation of the deeply localized “Pinch + Brush” neurons actually received inputs only from innocuous mechanical primary sensory neurons. Many heat-responsive neurons also responded to pinch stimulation (Fig. 6H, J, M), presumably because TRPV1, activated by noxious heat, is mainly expressed in nociceptive C fibers [44], [45]. In contrast, “Pinch + Brush + Heat” neurons and some subpopulation of “Pinch + Brush” neurons may receive inputs from both nociceptive and innocuous mechanical primary sensory neurons independently, previously described as wide dynamic range neurons [46]. It is likely that SDH interneurons relaying sensory signals between shallow and deep laminae play critical roles for the convergence of innocuous and nociceptive information. Excitatory interneurons with longer dorsoventral processes, such as vertical cells may be involved in such a trans-laminar neuronal network [35].

### Rostrocaudal propagation of sensory information in the SDH

In the present study, we examined calcium responses of large ensembles of SDH neurons following pinch stimulation toward different skin points; and our data revealed that cutaneous pinch stimulation evoked activities of SDH neurons localized rostrocaudally far beyond the central projection field of primary sensory neurons responsible for the stimulated skin point. Consistent with our finding, Bullitt reported that pinch stimulation to the lateral hip of the rat induces an increase in the number of *c-fos*-positive SDH neurons localized as far rostrally as T12 and as far caudally as L6 [47]. It is possible that the preferential rostrocaudal orientation of the dendritic arborization of SDH interneurons may partly account for the efficient propagation of primary sensory inputs along the rostrocaudal axis in the SDH [35]. Majority of P2- and P3-responsive SDH neurons were also sensitive to P1 stimulation (Fig. 9C, D), raising the possibility that pinch-responsive SDH neurons in the neighboring rostrocaudal area are interconnected by the excitatory neuronal network.

Our findings raise the question of the biological significance of the rostrocaudal expansion of SDH neuronal responses to focal sensory stimulation. A simple explanation is that propagation of sensory information in the SDH might be indispensable for amplification of sensory signal originating from a small area of the skin. Alternatively, this rostrocaudal propagation of sensory responses in the SDH might be involved in modulation of perceived sensory information in ways other than amplification. Treatment with GABA and glycine inhibitors expands the receptive field of cutaneous stimulation [9], [10], raising the possibility that inhibitory interneurons are used to spatially confine sensory perception. We may envisage that the propagation of neuronal responses in the SDH as revealed in this study might be recruited to further activate inhibitory interneurons across a wider rostrocaudal area, which eventually result in sharpening of the somatotopic precision of sensory information. *In vivo* patch-clamp recordings of medullary dorsal horn neurons demonstrated that the inhibitory receptive field of nociceptive neurons is wider than the excitatory receptive field [48]. Taken together, the most likely scenario is that the excitatory neuronal network has a potential to propagate sensory information to wider area along rostrocaudal axis but that this excitatory signal may be eventually suppressed by a stronger inhibitory neuronal network. In support of this idea, P2 and P3 stimulation evoked poor calcium transients of SDH neurons in the shallow lamina, which includes most projection neurons in the SDH.

### Future perspective

Analysis of three-dimensional distribution of neuronal activity maps in response to cutaneous sensory stimulation provides a clue to understand how primary sensory inputs propagate in the SDH through interneurons. Our next question is how each subtype of SDH interneurons contributes to the neuronal processing of sensory information. Expression of calcium indicators under the control of gene-specific promoters allows measurement of neuronal subtype-specific calcium responses [49]. Furthermore, *in vivo* calcium imaging in combination with pharmacological inhibition of SDH interneurons should clarify the significance of the global neuronal activity pattern of the SDH.

## Supporting Information

Figure S1 BrdU labeling. 5′-bromo-2′-deoxyuridine (BrdU) (Sigma) was intraperitoneally injected into pregnant ICR mice at E10.5, E11.5, E12.5, and E13.5. Transverse sections were prepared from the thoracic spinal cord of the labeled mice at P21. The sections were immunostained with rat anti-BrdU monoclonal antibody (Serotec) and mouse anti-NeuN antibody (Millipore). Scale bar, 200 µm.
(TIF)

Figure S2 Identity of EGFP-expressing cells in the SDH. *pCAG-EGFP* was electroporated into the spinal cord at E12.5, and transverse sections were prepared from the lumbar spinal cord of the electroporated mice at P21. The sections were immunostained with anti-NeuN (upper) or anti-GFAP (lower) antibody. EGFP fluorescence and immunofluorescence of anti-NeuN and anti-GFAP are shown. Scale bar, 100 µm.
(TIF)

Table S1 Number of pinch-, brush-, and heat-responsive SDH neurons. Recorded neurons are classified into 3 groups the depth from the dorsal edge of the grey matter: 0–50 µm, 50–100 µm, and 100–150 µm. The number of recorded and responsive neurons within each depth is shown (8 mice).
(XLS)

Table S2 Classification of responsive neurons by the amplitude of calcium transients. Recorded neurons described in Table S1 were further classified by the amplitude of calcium transients (ΔR/R). The lamina distribution pattern of responsive neurons within each category is shown (8 mice).
(XLS)

Table S3 Activation of SDH neurons in response to pinch stimulation applied to P1, P2, and P3. The number of neurons in response to pinch stimulation applied to P1, P2, and P3 is shown (14 mice).
(XLS)

Table S4 P2- and P3-evoked calcium transients in P1-responsive neurons. Among recorded neurons described in Table S3, 92 cells showed a strong response (ΔR/R>45%). Calcium transients following stimulation to P2 and P3 were analyzed, and peak amplitude of P2 and P3 stimulation relative to P1 stimulation among these 92 cells is shown.
(XLS)

Table S5 The lamina distribution of SDH neurons in response to pinch stimulation applied to P1, P2, and P3. Recorded neurons are classified into 3 groups according to the depth from the dorsal edge of the grey matter: 0–50 µm, 50–100 µm, and 100–150 µm. The number of responsive neurons among each group is shown (11 mice).
(XLS)

## References

[1] Todd AJ (2010) Neuronal circuitry for pain processing in the dorsal horn. Nat Rev Neurosci 11: 823–836. 21068766 10.1038/nrn2947 PMC3277941

[2] Yaksh TL (1989) Behavioral and autonomic correlates of the tactile evoked allodynia produced by spinal glycine inhibition: effects of modulatory receptor systems and excitatory amino acid antagonists. Pain 37: 111–123. 2542867 10.1016/0304-3959(89)90160-7

[3] Sherman SE, Loomis CW (1994) Morphine insensitive allodynia is produced by intrathecal strychnine in the lightly anesthetized rat. Pain 56: 17–29. 8159438 10.1016/0304-3959(94)90146-5

[4] Sorkin LS, Puig S, Jones DL (1998) Spinal bicuculline produces hypersensitivity of dorsal horn neurons: effects of excitatory amino acid antagonists. Pain 77: 181–190. 9766836 10.1016/S0304-3959(98)00094-3

[5] Ross SE, Mardinly AR, McCord AE, Zurawski J, Cohen S, et al (2010) Loss of inhibitory interneurons in the dorsal spinal cord and elevated itch in Bhlhb5 mutant mice. Neuron 65: 886–898. 20346763 10.1016/j.neuron.2010.02.025 PMC2856621

[6] Wang X, Zhang J, Eberhart D, Urban R, Meda K, et al (2013) Excitatory superficial dorsal horn interneurons are functionally heterogeneous and required for the full behavioral expression of pain and itch. Neuron 78: 312–324. 23622066 10.1016/j.neuron.2013.03.001 PMC3700415

[7] Xu Y, Lopes C, Wende H, Guo Z, Cheng L, et al (2013) Ontogeny of excitatory spinal neurons processing distinct somatic sensory modalities. J Neurosci 33: 14738–14748. 24027274 10.1523/JNEUROSCI.5512-12.2013 PMC3771039

[8] Sandkühler J (2009) Models and mechanisms of hyperalgesia and allodynia. Physiol Rev 89: 707–758. 19342617 10.1152/physrev.00025.2008

[9] Yokota T, Nishikawa N, Nishikawa Y (1979) Effects of strychnine upon different classes of trigeminal subnucleus caudalis neurons. Brain Res 168: 430–434. 109168 10.1016/0006-8993(79)90188-4

[10] Yokota T, Nishikawa Y (1979) Action of picrotoxin upon trigeminal subnucleus caudalis neurons in the monkey. Brain Res 171: 369–373. 111773 10.1016/0006-8993(79)90345-7

[11] Grienberger C, Konnerth A (2012) Imaging calcium in neurons. Neuron 73: 862–885. 22405199 10.1016/j.neuron.2012.02.011

[12] Ikeda H, Stark J, Fischer H, Wagner M, Drdla R, et al (2006) Synaptic amplifier of inflammatory pain in the spinal dorsal horn. Science 312: 1659–1662. 16778058 10.1126/science.1127233

[13] Drdla R, Gassner M, Gingl E, Sandkühler J (2009) Induction of synaptic long-term potentiation after opioid withdrawal. Science 325: 207–210. 19590003 10.1126/science.1171759

[14] Johannssen HC, Helmchen F (2010) In vivo Ca2+ imaging of dorsal horn neuronal populations in mouse spinal cord. J Physiol 588: 3397–3402. 20660563 10.1113/jphysiol.2010.191833 PMC2988506

[15] Johannssen HC, Helmchen F (2013) Two-photon imaging of spinal cord cellular networks. Exp. Neurol. 242: 18–26. 10.1016/j.expneurol.2012.07.014 22849822

[16] Laffray S, Pagès S, Dufour H, De Koninck P, De Koninck Y, et al (2011) Adaptive movement compensation for in vivo imaging of fast cellular dynamics within a moving tissue. PLoS One 6: e19928. 21629702 10.1371/journal.pone.0019928 PMC3101223

[17] Saba R, Nakatsuji N, Saito T (2003) Mammalian BarH1 confers commissural neuron identity on dorsal cells in the spinal cord. J Neurosci 23: 1987–1991. 12657654 10.1523/JNEUROSCI.23-06-01987.2003 PMC6742033

[18] Horikawa K, Yamada Y, Matsuda T, Kobayashi K, Hashimoto M, et al (2010) Spontaneous network activity visualized by ultrasensitive Ca(2+) indicators, yellow Cameleon-Nano. Nat Methods. 7: 729–732. 10.1038/nmeth.1488 20693999

[19] Hatanaka Y, Murakami F (2002) In vitro analysis of the origin, migratory behavior, and maturation of cortical pyramidal cells. J Comp Neurol 454: 1–14. 12410614 10.1002/cne.10421

[20] Zhu Y, Matsumoto T, Mikami S, Nagasawa T, Murakami F (2009) SDF1/CXCR4 signalling regulates two distinct processes of precerebellar neuronal migration and its depletion leads to abnormal pontine nuclei formation. Development 136: 1919–1928. 19429788 10.1242/dev.032276

[21] Nishida K, Nakayama K, Yoshimura S, Murakami F (2011) Role of Neph2 in pontine nuclei formation in the developing hindbrain. Mol Cell Neurosci 46: 662–670. 21241806 10.1016/j.mcn.2011.01.007

[22] Niwa H, Yamamura K, Miyazaki J (1991) Efficient selection for high-expression transfectants with a novel eukaryotic vector. Gene 108: 193–199. 1660837 10.1016/0378-1119(91)90434-d

[23] Dai JX, Hu ZL, Shi M, Guo C, Ding YQ (2008) Postnatal ontogeny of the transcription factor Lmx1b in the mouse central nervous system. J Comp Neurol 509: 341–355. 18512225 10.1002/cne.21759

[24] Rexed B (1952) The cytoarchitectonic organization of the spinal cord in the cat. J Comp Neurol 96: 414–495. 14946260 10.1002/cne.900960303

[25] Cheng L, Arata A, Mizuguchi R, Qian Y, Karunaratne A, et al (2004) Tlx3 and Tlx1 are post-mitotic selector genes determining glutamatergic over GABAergic cell fates. Nat Neurosci 7: 510–517. 15064766 10.1038/nn1221

[26] Luu B, Ellisor D, Zervas M (2011) The lineage contribution and role of Gbx2 in spinal cord development. PLoS One 6: e20940. 21698205 10.1371/journal.pone.0020940 PMC3116860

[27] Albuquerque C, Lee CJ, Jackson AC, MacDermott AB (1999) Subpopulations of GABAergic and non-GABAergic rat dorsal horn neurons express Ca2+-permeable AMPA receptors. Eur J Neurosci 11: 2758–2766. 10457172 10.1046/j.1460-9568.1999.00691.x

[28] Sardella TC, Polgár E, Watanabe M, Todd AJ (2011) A quantitative study of neuronal nitric oxide synthase expression in laminae I–III of the rat spinal dorsal horn. Neuroscience 192: 708–720. 21763759 10.1016/j.neuroscience.2011.07.011 PMC3183229

[29] Takahashi Y, Chiba T, Sameda H, Ohtori S, Kurokawa M, et al (2002) Organization of cutaneous ventrodorsal and rostrocaudal axial lines in the rat hindlimb and trunk in the dorsal horn of the spinal cord. J Comp Neurol 445: 133–144. 11891658 10.1002/cne.10158

[30] Li L, Rutlin M, Abraira VE, Cassidy C, Kus L, et al (2011) The functional organization of cutaneous low-threshold mechanosensory neurons. Cell 147: 1615–1627. 22196735 10.1016/j.cell.2011.11.027 PMC3262167

[31] Light AR, Perl ER (1979) Spinal termination of functionally identified primary afferent neurons with slowly conducting myelinated fibers. J Comp Neurol 186: 133–150. 109477 10.1002/cne.901860203

[32] Sugiura Y, Lee CL, Perl ER (1986) Central projections of identified, unmyelinated (C) afferent fibers innervating mammalian skin. Science 234: 358–361. 3764416 10.1126/science.3764416

[33] Lorenzo LE, Ramien M, St Louis M, De Koninck Y, Ribeiro-da-Silva A (2008) Postnatal changes in the Rexed lamination and markers of nociceptive afferents in the superficial dorsal horn of the rat. J Comp Neurol 508: 592–604. 18383051 10.1002/cne.21691

[34] Tominaga M, Caterina MJ, Malmberg AB, Rosen TA, Gilbert H, et al (1998) The cloned capsaicin receptor integrates multiple pain-producing stimuli. Neuron 21: 531–543. 9768840 10.1016/s0896-6273(00)80564-4

[35] Grudt TJ, Perl ER (2002) Correlations between neuronal morphology and electrophysiological features in the rodent superficial dorsal horn. J Physiol 540: 189–207. 11927679 10.1113/jphysiol.2001.012890 PMC2290200

[36] Furue H, Narikawa K, Kumamoto E, Yoshimura M (1999) Responsiveness of rat substantia gelatinosa neurones to mechanical but not thermal stimuli revealed by in vivo patch-clamp recording. J Physiol 521 Pt 2: 529–535. 10.1111/j.1469-7793.1999.00529.x PMC2269671 10581321

[37] Light AR, Willcockson HH (1999) Spinal laminae I–II neurons in rat recorded in vivo in whole cell, tight seal configuration: properties and opioid responses. J Neurophysiol 82: 3316–3326. 10601463 10.1152/jn.1999.82.6.3316

[38] Chen TW, Wardill TJ, Sun Y, Pulver SR, Renninger SL, et al (2013) Ultrasensitive fluorescent proteins for imaging neuronal activity. Nature 499: 295–300. 23868258 10.1038/nature12354 PMC3777791

[39] Woolf CJ, Fitzgerald M (1983) The properties of neurones recorded in the superficial dorsal horn of the rat spinal cord. J Comp Neurol 221: 313–328. 6197429 10.1002/cne.902210307

[40] Narikawa K, Furue H, Kumamoto E, Yoshimura M (2000) In vivo patch-clamp analysis of IPSCs evoked in rat substantia gelatinosa neurons by cutaneous mechanical stimulation. J Neurophysiol 84: 2171–2174. 11024105 10.1152/jn.2000.84.4.2171

[41] Yasaka T, Tiong SY, Hughes DI, Riddell JS, Todd AJ (2010) Populations of inhibitory and excitatory interneurons in lamina II of the adult rat spinal dorsal horn revealed by a combined electrophysiological and anatomical approach. Pain 151: 475–488. 20817353 10.1016/j.pain.2010.08.008 PMC3170912

[42] Kato G, Kawasaki Y, Koga K, Uta D, Kosugi M, et al (2009) Organization of intralaminar and translaminar neuronal connectivity in the superficial spinal dorsal horn. J Neurosci 29: 5088–5099. 19386904 10.1523/JNEUROSCI.6175-08.2009 PMC2777732

[43] Graham BA, Brichta AM, Callister RJ (2004) In vivo responses of mouse superficial dorsal horn neurones to both current injection and peripheral cutaneous stimulation. J Physiol 561: 749–763. 15604230 10.1113/jphysiol.2004.072645 PMC1665382

[44] Michael GJ, Priestley JV (1999) Differential expression of the mRNA for the vanilloid receptor subtype 1 in cells of the adult rat dorsal root and nodose ganglia and its downregulation by axotomy. J Neurosci 19: 1844–1854. 10024368 10.1523/JNEUROSCI.19-05-01844.1999 PMC6782176

[45] Kobayashi K, Fukuoka T, Obata K, Yamanaka H, Dai Y, et al (2005) Distinct expression of TRPM8, TRPA1, and TRPV1 mRNAs in rat primary afferent neurons with adelta/c-fibers and colocalization with trk receptors. J Comp Neurol 493: 596–606. 16304633 10.1002/cne.20794

[46] Le Bars D (2002) The whole body receptive field of dorsal horn multireceptive neurones. Brain Res Brain Res Rev 40: 29–44. 12589904 10.1016/s0165-0173(02)00186-8

[47] Bullitt E (1991) Somatotopy of spinal nociceptive processing. J Comp Neurol 312: 279–290. 1748733 10.1002/cne.903120210

[48] Kato G, Kosugi M, Mizuno M, Strassman AM (2011) Separate inhibitory and excitatory components underlying receptive field organization in superficial medullary dorsal horn neurons. J Neurosci 31: 17300–17305. 22114296 10.1523/JNEUROSCI.4474-11.2011 PMC3241980

[49] Avraham O, Hadas Y, Vald L, Zisman S, Schejter A, et al (2009) Transcriptional control of axonal guidance and sorting in dorsal interneurons by the Lim-HD proteins Lhx9 and Lhx1. Neural Dev 4: 21. 19545367 10.1186/1749-8104-4-21 PMC2704203

