# Supplementary figures and images for: Three-Dimensional Distribution of Sensory Stimulation-Evoked Neuronal Activity of Spinal Dorsal Horn Neurons Analyzed by In Vivo Calcium Imaging
Source: PLoS One. 2014 Aug 6;9(8):e103321. doi: 10.1371/journal.pone.0103321 (PMC4123881; doi:10.1371/journal.pone.0103321)

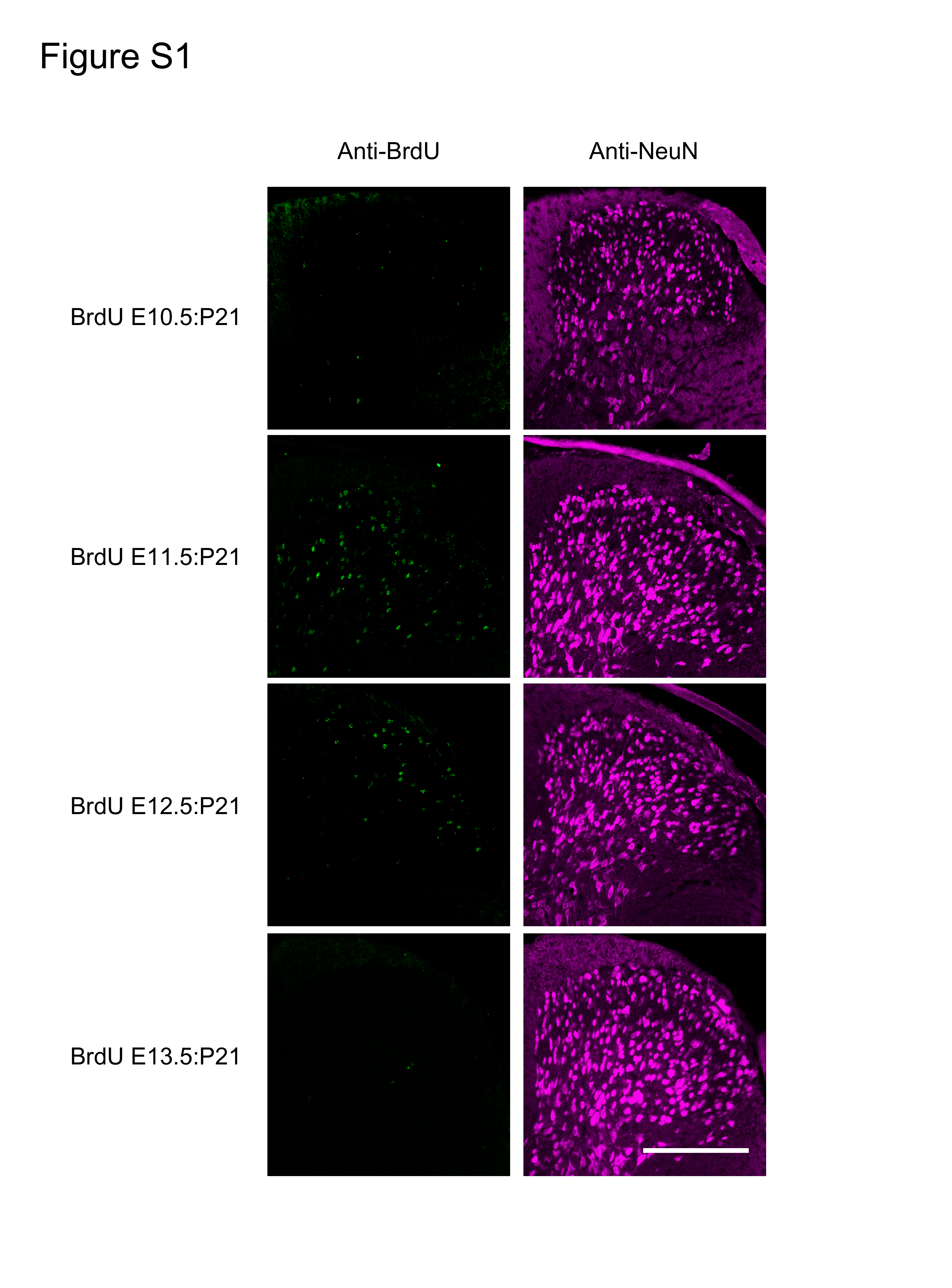

Supplement: Figure S1 — BrdU labeling. 5′-bromo-2′-deoxyuridine (BrdU) (Sigma) was intraperitoneally injected into pregnant ICR mice at E10.5, E11.5, E12.5, and E13.5. Transverse sections were prepared from the thoracic spinal cord of the labeled mice at P21. The sections were immunostained with rat anti-BrdU monoclonal antibody (Serotec) and mouse anti-NeuN antibody (Millipore). Scale bar, 200 µm. (TIF) [file pone.0103321.s001.tif]

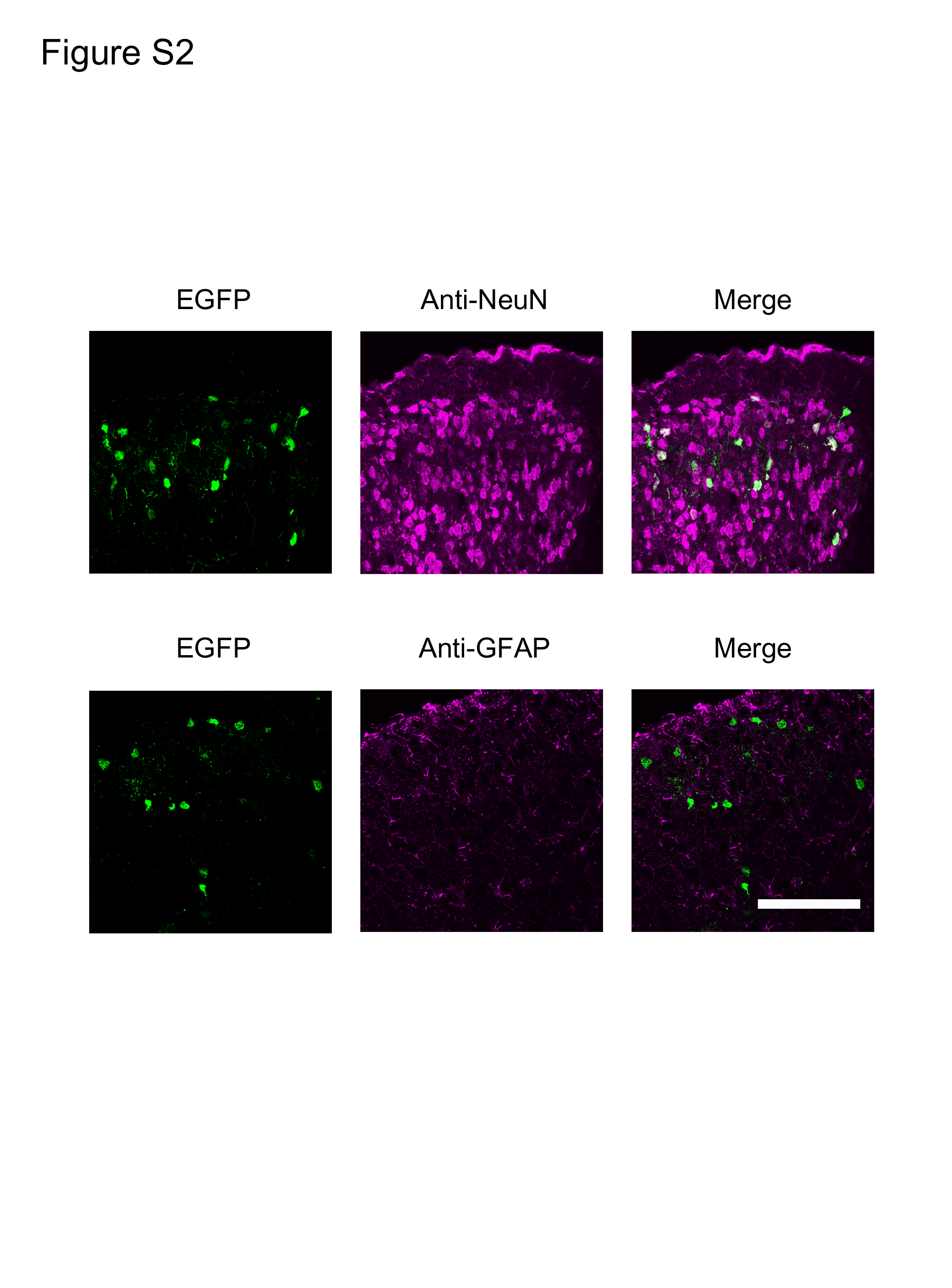

Supplement: Figure S2 — Identity of EGFP-expressing cells in the SDH. pCAG-EGFP was electroporated into the spinal cord at E12.5, and transverse sections were prepared from the lumbar spinal cord of the electroporated mice at P21. The sections were immunostained with anti-NeuN (upper) or anti-GFAP (lower) antibody. EGFP fluorescence and immunofluorescence of anti-NeuN and anti-GFAP are shown. Scale bar, 100 µm. (TIF) [file pone.0103321.s002.tif]
